# Immunogenic cell death as driver of autoimmunity in granulomatosis with polyangiitis

**DOI:** 10.3389/fimmu.2022.1007092

**Published:** 2022-10-06

**Authors:** Christoph Brieske, Peter Lamprecht, Anja Kerstein-Staehle

**Affiliations:** Department of Rheumatology and Clinical Immunology, University of Lübeck, Lübeck, Germany

**Keywords:** immunogenic cell death, autoimmunity, neutrophils, vasculitis, granulomatosis with polyangiitis, damage-associated molecular pattern (DAMP)

## Abstract

Cell death and dysregulated clearance of dead cells play essential roles in the induction of chronic inflammatory processes and autoimmune diseases. Granulomatosis with polyangiitis (GPA), a neutrophil-driven autoimmune disorder, is characterized by necrotizing inflammation predominantly of the respiratory tract and an anti-neutrophil cytoplasmic autoantibody (ANCA)-associated systemic necrotizing vasculitis. Defective regulation of neutrophil homeostasis and cell death mechanisms have been demonstrated in GPA. Disturbed efferocytosis (*i.e*., phagocytosis of apoptotic neutrophils by macrophages) as well as cell death-related release of damage-associated molecular patterns (DAMP) such as high mobility group box 1 (HMGB1) contribute to chronic non-resolving inflammation in GPA. DAMP have been shown to induce innate as well as adaptive cellular responses thereby creating a prerequisite for the development of pathogenic autoimmunity. In this review, we discuss factors contributing to as well as the impact of regulated cell death (RCD) accompanied by DAMP-release as early drivers of the granulomatous tissue inflammation and autoimmune responses in GPA.

## Introduction

Granulomatosis with polyangiitis (GPA) is an anti-neutrophil cytoplasmic autoantibody (ANCA)-associated vasculitis (AAV). GPA is a rare and severe autoimmune disease characterized by necrotizing granulomatous inflammation localized predominantly in the upper and/or lower respiratory tract and ANCA-induced systemic necrotizing small vessel vasculitis (1). Its multifactorial etiopathogenesis is autoantigen- and pathogen-triggered, but still not fully understood. In more detail, predisposing genetic factors, environmental impact as well as immune-modulating pathways within the tissue microenvironment serve as initial trigger for the recruitment and activation of extravascular primed neutrophils forming neutrophil-rich microabscesses (2, 3). These microabcesses over time begin to form ill-defined extravascular granulomas accompanied by large areas of geographic necrosis and typical innate immune cell infiltrates, which, together with the necrotizing vasculitis, mark the classical histomorphologic triad of GPA (4, 5). A central pathophysiological feature related to the necrotizing, granulomatous inflammation in GPA is the dysregulation of neutrophil homeostasis on genetic, molecular, and cellular level that is associated with an increased and sustained exposure of proteinase 3 (PR3), the main target antigen of ANCA in GPA. In particular, cell death mechanisms (dysregulated apoptosis, necrosis, necroptosis, and NETosis) lead to the release of damage-associated molecular patterns (DAMP), contribute to increased availability of (altered) PR3 and activate the local adaptive immune system favoring the formation of ectopic or tertiary lymphoid structures (ELS or TLS) (3). Cellular defects, *e.g.* in the regulation of the inhibitory immune checkpoint molecule programmed death-ligand 1 (PD-L1) in monocytes promote the development of highly activated T cells in AAV (6). Along with this, the continuous and/or altered presentation of PR3 to highly activated T cells accompanied by local inflammation-induced B and plasma cell survival, this sets the prerequisite for the loss of tolerance towards PR3 with subsequent production of ANCA in GPA (7, 8). Tissue damage occurs as a result of ANCA-induced systemic vasculitis and inflammation-mediated local tissue destruction predominantly in the upper respiratory tract (1, 9). Since therapeutic approaches are mainly symptomatic, *e.g.* immunosuppression, B cell depletion or complement-inhibition (10, 11), a future goal would be an intervention in early pathophysiological events from which the loss of tolerance against PR3 emerges. Therefore, in this review we discuss the role of immunogenic cell death as an early pathophysiological event in GPA involved in the development of the granulomatous inflammation possibly before the autoimmune response occurs.

## Cell death mechanisms

Cell death is a ubiquitous process that can be either immunostimulatory or immunologically silent depending on the activated cellular components and on the signals from the surrounding tissue. On this basis, cell death can generally be divided into unregulated necrosis and several forms of regulated cell death (RCD). RCD is further differentiated into caspase-dependent and independent forms or based on the immunogenic outcome. Caspase-dependent forms of RCD include apoptosis, which is characterized by nuclear fragmentation (karyorrhexis), cytoplasmic shrinkage, chromatin condensation (pyknosis) and plasma membrane blebbing releasing small cell vesicles with intact cell membranes. Apoptosis is usually immunogenic silent under physiological conditions (12). Apoptosis is initiated either *via* intra- and extracellular perturbations (intrinsic apoptosis) or *via* death-receptor activation (extrinsic apoptosis) leading to activation of a cascade of several caspases with caspase 3 as the main executioner caspase (12). In addition to apoptosis, pyroptosis is another caspase-dependent cell death pathway, that has important functions in host defense and inflammation. Pyroptosis relies on the inflammatory activation of several caspases, such as caspase 1 or 11, that can be part of a multiprotein platform known as the inflammasome. Inflammasome-dependent pyroptosis controls the production of important pro-inflammatory cytokines such as interleukin-1β (IL-1β) and IL-18 and critically depends on the formation of plasma membrane pores by members of the gasdermin protein family (13).

A caspase-independent RCD is necroptosis, which has become an increasing focus of inflammation research in recent years (14, 15). In contrast to previous assumptions that necrosis occurs as a purely uncontrolled event due to physiochemical stress, osmotic shock or freeze/thaw, necroptosis is a form of regulated necrosis. Necroptosis is a receptor-mediated RCD characterized by a complex interplay of receptor-interacting serine/threonine protein kinase (RIPK) 1, RIPK3, and mixed lineage kinase domain-like pseudokinase (MLKL), among others. Necroptosis is associated with loss of membrane integrity and the release of several DAMP (15, 16). Triggered similarly to apoptosis, necroptosis can be induced by activation of various death receptors of the tumor necrosis factor (TNF) superfamily, but also toll-like receptor (TLR) 3, TLR4, or interferon receptors. In fact, the initial steps to induce apoptosis or necroptosis are overlapping. However, the decision as to which of the two types of cell death is ultimately executed depends on the cell type, the nature of the stimulus, and the activity or inhibition of other intracellular factors (14, 17).

A more atypical form of cell death related to neutrophils is NETosis, that is morphologically distinct from apoptosis and necrosis (18). During NETosis, neutrophils extrude in a reactive oxygen species (ROS)-dependent manner so called neutrophil extracellular traps (NET), *i.e*., long strands of nuclear DNA carrying cytoplasmic and granular proteins with antimicrobial and proinflammatory characteristics, initially described as a defense mechanism against bacteria (19).

## Damage-associated molecular patterns

Cell death is a process important during normal cell turnover, elimination of pathogens or promotion of wound healing. The question is, therefore, how the immune system determines whether cell death is immunogenic or tolerogenic. In this context, Polly Matzinger introduced the concept of the danger model, stating that an immune response is induced by endogenous and exogenous danger signals from injured or stressed cells (20). According to this concept, autoimmunity would arise from altered or increased presentation of antigen in the presence of persistent or chronic release of danger signals (21). These danger signals have been termed damage-associated molecular patterns (DAMP), referring to the ability of DAMP to elicit similar responses and to utilize the same receptors as their exogenous counterparts, the pathogen-associated molecular patterns (PAMP) (22, 23). The growing list of DAMP include nuclear, mitochondrial, or cytosolic factors with physiological roles within the cell that converts into danger signals when released to the extracellular milieu during inflammation or cell death (24). DAMP are then sensed by a broad range of receptors, e.g. Toll-like receptors (TLR) or NOD-like receptors (NLR) to alert the immune system by inducing immune cell migration, increasing phagocytosis by macrophages and dendritic cells (DC), stimulating the production of pro-inflammatory cytokines or contributing to the maturation of DC (25, 26). Several DAMP are already well characterized regarding the associated type of cell death and their role in the immune system (23).

## Cell death and danger signaling in autoimmune diseases

For the maintenance of tissue homeostasis, a balance between cell proliferation, differentiation and death is crucial. In this regard, the immune system is routinely exposed to dead cells during normal cell turnover, injury, and infection. However, dysregulation of cell death mechanisms such as increased cell death rate, (genetic) alterations of cell death-related molecules or defective clearance of dead cells accompanied by massive release of DAMP can lead to uncontrolled excessive and/or prolonged inflammatory responses with subsequent tissue damage, thereby contributing to the pathogenesis of several pathologies, such as autoimmune diseases.

Apoptosis is a cell death program mostly related to non-inflammatory outcomes and likely to take major role in the maintenance of homeostasis by silently eliminating unwanted or damaged cells. However, defects in mechanisms of apoptotic cell clearance are linked to autoimmunity disorders, including systemic lupus erythematosus (SLE) and rheumatoid arthritis (RA), likely due to the increased risk of loss of cell integrity with the consequent release of DAMP and increased availability of circulating self-antigens (23, 27).

Necroptosis has a profound proinflammatory effect, described in various inflammatory and autoimmune disorders, such as inflammatory bowel disease (IBD), RA, multiple sclerosis (MS) and SLE. Increased expression or phosphorylation of RIPK3 or MLKL and reduced expression of Caspase 8, have been detected in pathological samples (15, 28).

A role for inflammasome activation during pyroptosis in autoimmune diseases is likely, considering the wide spectrum of endogenous danger signals that activate NLR and the role of IL-1β and IL-18 in shaping adaptive immunity. More precisely, IL-1β and IL-18 amplify T and B cell responses and might serve as a crucial link translating NLR activation into adaptive immune responses. SNP in NLRP3 gene and an effect of pharmacological IL-1 blockade underline the clinical relevance for pyroptosis in autoimmunity, *e.g*., in SLE, MS and type-1 diabetes (T1D) (29).

NET formation has been identified as a link between innate and adaptive immune responses in autoimmunity. Autoantigens including neutrophil granular proteins and post-translationally modified proteins, *e.g.*, citrullinated proteins, localize on NET. NET promote inflammation and provide stimuli to dendritic cells and can directly activate B and T cells, thereby potentiating adaptive autoimmune responses and induction autoreactivity against NET-associated antigens. NET have been detected *in vivo* in SLE, RA, IBD and AAV (18, 30).

As mentioned above, necrosis and RCD is accompanied by cell death-related release of DAMP. The best characterized and clinically relevant DAMP include high-mobility group box 1 (HMGB1), S100 proteins S100A8/A9/A12, heat shock proteins HSP60 and HSP70, β-defensins, and the cathelicidin LL-37, turning them into attractive therapeutic targets of chronic inflammatory and autoimmune diseases (31).

## Immunogenic cell death in GPA

Necrotizing granulomatous inflammation is rich in neutrophils. In early lesions, numerous microabscesses formed by neutrophils are conspicuous, which presumably represent the starting point of the later necrosis areas. While ANCA-induced vasculitis is well studied, the pathogenetic mechanisms underlying necrotizing inflammation in GPA are poorly understood. It has been postulated that defects in the cell death machinery may contribute to the inflammatory response and autoimmunity in GPA (2, 3).

In this context, apoptosis has been the most and best studied cell death in GPA. On genetic level, an association with apoptosis-related genes was found in GPA, suggesting a shift in the balance of apoptosis (32). On cellular level, dysregulation of apoptosis in isolated neutrophils has been observed. Prolonged survival and/or a delay in apoptosis rate bear the risk of insufficient elimination of apoptotic neutrophils, that may lead to secondary necrosis releasing the intracellular content including proteases, pro-inflammatory cytokines, DAMP, and potential autoantigens into the tissue (33, 34). There is evidence that the autoantigen PR3 itself plays an essential role in this context. On the one hand, PR3 was constitutively expressed at the plasma membrane of GPA neutrophils, and the percentage of neutrophils expressing PR3 was increased in GPA, demonstrating an abnormal increased availability of PR3 (35). On the other hand, PR3 co-externalized and interfered with “eat-me”-signals during apoptosis, thus impairing phagocytosis by macrophages (35). Moreover, we and others have shown that membrane expression of PR3 on apoptotic cells induced a pro-inflammatory response in macrophages and impaired their anti-inflammatory reprogramming following efferocytosis, indicating the impact of PR3 to alter the immunosuppressive effect of apoptotic cell efferocytosis and to promote sustained inflammation in GPA (35, 36).

In addition to (dysregulated) apoptosis, NET formation was initially observed in response to ANCA-stimulation in AAV including GPA (37). The authors further identified NET deposition in biopsies of inflamed kidney in active disease, demonstrating an *in vivo* relevance for NETosis in AAV. Since then, numerous studies have been conducted on the role of NET in AAV. In particular, NET can activate the alternative complement pathway with the production of C5a, that in turn primes and recruits more neutrophils, thereby amplifying the inflammatory process in AAV (38–40). Moreover, NET and NET-associated proteins were increased in the circulation of AAV patients, they contribute to vessel inflammation and act as a link between the innate and adaptive immune system by possessing a high immunogenicity and possibly promoting autoimmune responses against neutrophil components such as PR3 or MPO, thus inducing pathogenic ANCA production (41). In the context of GPA, PR3 shows unique structural and functional properties, in particular anchorage into the plasma membrane *via* a hydrophobic patch, which might render PR3 the favored autoantigen in GPA (42).

Furthermore, there is evidence that inflammasome-dependent and independent IL-1β processing and secretion is involved in GPA pathogenesis. IL-1β positive cells were found in renal biopsies from patients with AAV including GPA (43). As described above, processing of IL-1β in neutrophils and monocytes can be mediated by the NLRP3-inflammasome. In accordance with this, Factor H related protein 1 (FHR1) was found to be elevated in AAV serum. FHR1 was able to activate the NLRP3 inflammasome and IL-1β release from monocytes when bound to necrotic cells in AAV (44). In contrast, stimulation of human monocytes and neutrophils with monoclonal antibodies to PR3 or myeloperoxidase (MPO, the main autoantigen of microscopic polyangiitis, another AAV) or with human ANCA IgG, led to IL-1β generation and release in an inflammasome-independent manner. Instead, the authors of this study found that PR3 is the main protease involved in IL-1β maturation (45). In line with this, another study showed that neutrophils phagocytosing *Staphylococcus aureus* triggered a RIPK3-mediated activation of serine proteases in neutrophils independent of the NLRP3-inflammasome. Processing and secretion of IL-1β as well as cleavage of gasdermin D was PR3-dependent, leading to membrane pore formation and subsequent lytic cell death of neutrophils (46, 47). In addition, we identified a gene signature related to the inflammasome pathway (NLRP3, IL-1b, IL-18) as well as a significant enrichment of pathways consistent with immune responses triggered by *S. aureus* on the transcriptomic level in sorted T cells in GPA (48). *S. aureus* has been implicated in the induction and modulation of AAV including GPA. Studies have demonstrated that chronic nasal carriage of *S. aureus* was associated with higher relapse risk and endonasal activity in GPA (49, 50). Notably, *S. aureus* infection was linked to necroptosis in neutrophils and macrophages as well as with interference of efferocytosis, thereby contributing to inflammatory pathology (51, 52). Indeed, a functional link between RIP3-dependent necroptosis and ANCA-induced NET formation was discovered in murine MPO-AAV models as well as in human kidney biopsies of ANCA-associated necrotizing crescentic glomerulonephritis (30). These data suggest that infection-triggered necroptosis might be a relevant immunogenic cell death in GPA, as well.

## DAMP signaling in AAV

Cell death-related DAMP signaling has been described in several studies in AAV. One of the best characterized DAMP in this regard is HMGB1. HMGB1 is a ubiquitously expressed nuclear protein with structural function such as stabilizing nucleosome structure and DNA bending (53). During activation or cell death, HMGB1 can be released from various cells mediating inflammation and acting as endogenous adjuvant, thus inducing autoimmunity (54, 55). There in increasing evidence that HMGB1 could represent a biomarker for disease activity, might act as pro-inflammatory mediator as well as inducer of autoimmunity in GPA.

In this context, we and others have shown a correlation between elevated serum HMGB1 with both disease activity and pulmonary granuloma. Therefore, HMGB1 may be used as a marker of the burden of granulomatous inflammation in GPA (56–58). MPO-positive microparticles (MPO+MPs), that could originate from activated or apoptotic neutrophils, are increased in plasma from patients with AAV. HMGB1 expressing MPO+MPs were associated with disease activity in AAV (59). HMGB1 could prime neutrophils by increasing ANCA antigens translocation, and the primed neutrophils could be further induced by ANCA, resulting in the respiratory burst and degranulation in a TLR4- and RAGE-dependent manner (60). Furthermore, the interaction between HMGB1 and C5a plays an important role in ANCA-induced neutrophil activation. This was shown by inhibition of HMGB1 leading to a decreased C5a-mediated translocation of ANCA antigens, as well as ANCA-induced respiratory burst and degranulation of C5a-primed neutrophils (60). HMGB1 released by C5a-primed neutrophils can further induce ANCA antigen expression in an amplificatory manner, thus resulting in more intense activation of neutrophils (61). In addition, HMGB1 could enhance TLR9 expression in plasma cells and B cell proliferation and TLR9 expression in plasma cells was associated with disease activity in AAV (62). Plasma levels of HMGB1 correlated with endothelial cell activation in AAV patients. Further, HMGB1 amplified neutrophil activation, increased expression of intercellular adhesion molecules as well as the injury of glomerular endothelial cells in the presence of ANCA (63). The receptor of advanced glycation end products (RAGE), one of the main receptors of HMGB1, was highly expressed on cells infiltrating AAV kidney and lung biopsy tissue and mRNA expression was enhanced in PBMCs from active AAV patients (64).

These data demonstrate, that DAMP-signaling, here HMGB1, participates in neutrophil activation, B cell proliferation, endothelial cell activation, receptor-mediated inflammation as well as increased ANCA antigen translocation, thereby playing an essential role in inflammatory and autoimmune processes in GPA.

## Discussion

**Figure 1:**
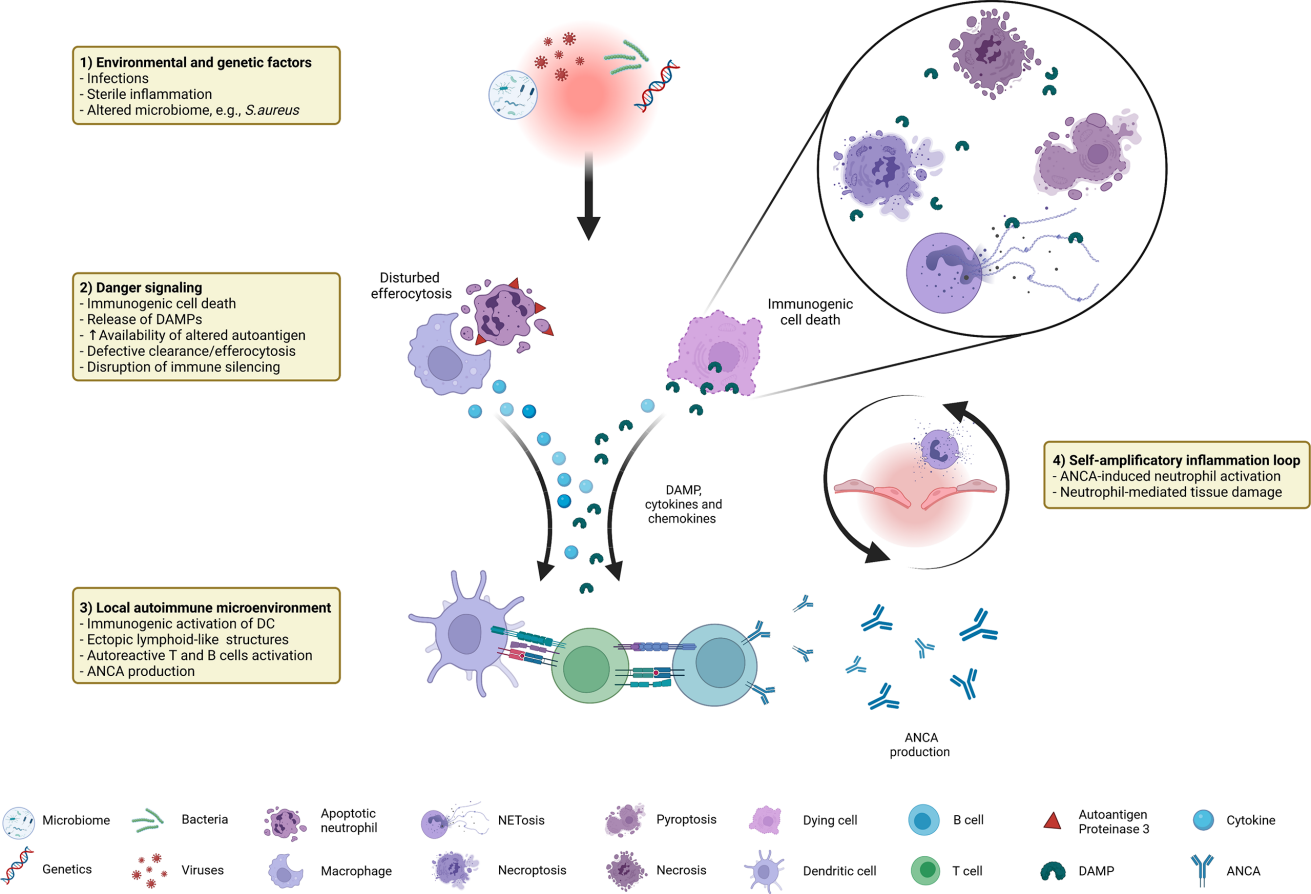
Immunogenic cell death in GPA at a glance: distinctive factors and mechanisms associated with immunogenic cell death contribute to autoimmune reactions in GPA.

In this review we provide an overview about factors contributing to and the consequences of immunogenic cell death in GPA. As summarized in **Figure 1**, a combination of predisposing genetic factors as well as exogenous and endogenous immune-stimulatory patterns such as infections (e.g., *S. aureus*), altered microbiome, and sterile inflammation serve as initial trigger for the recruitment and activation of extravascular primed neutrophils forming neutrophil-rich microabscesses. Immunogenic cell death (dysregulated apoptosis, pyroptosis, necroptosis, NETosis) leads to the release of DAMP, such as HMGB1, contributes to increased availability of the (altered) autoantigen PR3 and participates in the disruption of immune silencing due to disturbed efferocytosis. Concomitant pro-inflammatory cytokines, chemokines as well as complement drive recruitment of further immune cells, promoting the formation of inflammation-related ectopic lymphoid structures. Subsequent activation of the adaptive immune system set the prerequisite for the loss of tolerance against the autoantigen PR3 that is continuously displayed by high numbers of (dying) neutrophils and monocytes within the granulomatous lesion. ANCA induce systemic necrotizing vasculitis with further tissue damage, creating a self-amplificatory inflammation loop leading to chronification of inflammation (non-resolving inflammation) with tissue destruction and even organ failure. Therefore, a therapeutic intervention in cell death mechanisms could be a beneficial approach to prevent the chronification and autoimmune reactions at an early time point, possibly before the break of tolerance against PR3 occurs in GPA.

## Author contributions

CB and AK-S designed and wrote the manuscript. PL corrected and advised the writing process. All authors contributed to the article and approved the submitted version.

## Funding

Deutsche Forschungsgemeinschaft, Research Training Group 2633.

## Conflict of interest

The authors declare that the research was conducted in the absence of any commercial or financial relationships that could be construed as a potential conflict of interest.

## Publisher’s note

All claims expressed in this article are solely those of the authors and do not necessarily represent those of their affiliated organizations, or those of the publisher, the editors and the reviewers. Any product that may be evaluated in this article, or claim that may be made by its manufacturer, is not guaranteed or endorsed by the publisher.

## References

[1] Jennette JC Falk RJ. Pathogenesis of antineutrophil cytoplasmic autoantibody-mediated disease. Nat Rev Rheumatol (2014) 10:463–73. doi: 10.1038/nrrheum.2014.103 25003769

[2] Lamprecht P Kerstein A Klapa S Schinke S Karsten CM Yu X. Pathogenetic and clinical aspects of anti-neutrophil cytoplasmic autoantibody-associated vasculitides. Front Immunol (2018) 9:680. doi: 10.3389/fimmu.2018.00680 29686675 PMC5900791

[3] Müller A Krause B Kerstein-Stähle A Comdühr S Klapa S Ullrich S. Granulomatous inflammation in ANCA-associated vasculitis. Int J Mol Sci (2021) 22:6474. doi: 10.3390/ijms22126474 34204207 PMC8234846

[4] Kerstein A Holl-Ulrich K Müller A Riemekasten G Lamprecht P. Granulomatose mit polyangiitis. DMW - Dtsch Medizinische Wochenschrift (2017) 142:24–31. doi: 10.1055/s-0042-111610 28056474

[5] Holl-Ulrich K. Histopathology of systemic vasculitis. Pathologe (2010) 31:67–76. doi: 10.1007/s00292-009-1156-x 19533137

[6] Zeisbrich M Chevalier N Sehnert B Rizzi M Venhoff N Thiel J. CMTM6-deficient monocytes in ANCA-associated vasculitis fail to present the immune checkpoint PD-L1. Front Immunol (2021) 12:673912. doi: 10.3389/fimmu.2021.673912 34108971 PMC8183471

[7] Mueller A Brieske C Schinke S Csernok E Gross WL Hasselbacher K. Plasma cells within granulomatous inflammation display signs pointing towards autoreactivity and destruction in granulomatosis with polyangiitis. Arthritis Res Ther (2014) 16:R55. doi: 10.1186/ar4490 24555783 PMC3978674

[8] Weppner G Ohlei O Hammers CM Holl-Ulrich K Voswinkel J Bischof J. *In situ* detection of PR3-ANCA+ b cells and alterations in the variable region of immunoglobulin genes support a role of inflamed tissue in the emergence of auto-reactivity in granulomatosis with polyangiitis. J Autoimmun (2018) 93:89–103. doi: 10.1016/j.jaut.2018.07.004 30054207

[9] Kitching AR Anders H-J Basu N Brouwer E Gordon J Jayne DR. ANCA-associated vasculitis. Nat Rev Dis Prim (2020) 6:71. doi: 10.1038/s41572-020-0204-y 32855422

[10] Schönermarck U Gross WL de Groot K. Treatment of ANCA-associated vasculitis. Nat Rev Nephrol (2014) 10:25–36. doi: 10.1038/nrneph.2013.225 24189648

[11] Jayne DRW Merkel PA Schall TJ Bekker P. Avacopan for the treatment of ANCA-associated vasculitis. N Engl J Med (2021) 384:599–609. doi: 10.1056/NEJMoa2023386 33596356

[12] Galluzzi L Vitale I Aaronson SA Abrams JM Adam D Agostinis P. Molecular mechanisms of cell death: Recommendations of the nomenclature committee on cell death 2018. Cell Death Differ (2018) 25:486–541. doi: 10.1038/s41418-017-0012-4 29362479 PMC5864239

[13] Yu P Zhang X Liu N Tang L Peng C Chen X. Pyroptosis: mechanisms and diseases. Signal Transduct Target Ther (2021) 6:128. doi: 10.1038/s41392-021-00507-5 33776057 PMC8005494

[14] Pasparakis M Vandenabeele P. Necroptosis and its role in inflammation. Nature (2015) 517:311–20. doi: 10.1038/nature14191 25592536

[15] Molnár T Mázló A Tslaf V Szöllősi AG Emri G Koncz G. Current translational potential and underlying molecular mechanisms of necroptosis. Cell Death Dis (2019) 10:860. doi: 10.1038/s41419-019-2094-z 31719524 PMC6851151

[16] Kaczmarek A Vandenabeele P Krysko DV. Necroptosis: The release of damage-associated molecular patterns and its physiological relevance. Immunity (2013) 38:209–23. doi: 10.1016/j.immuni.2013.02.003 23438821

[17] Han J Zhong C-Q Zhang D-W. Programmed necrosis: backup to and competitor with apoptosis in the immune system. Nat Immunol (2011) 12:1143–9. doi: 10.1038/ni.2159 22089220

[18] Skopelja-Gardner S Jones JD Rigby WFC. “NETtling” the host: Breaking of tolerance in chronic inflammation and chronic infection. J Autoimmun (2018) 88:1–10. doi: 10.1016/j.jaut.2017.10.008 29100671

[19] Brinkmann V Reichard U Goosmann C Fauler B Uhlemann Y Weiss DS. Neutrophil extracellular traps kill bacteria. Sci (80- ) (2004) 303:1532–5. doi: 10.1126/science.1092385 15001782

[20] Matzinger P. Tolerance, danger, and the extended family. Annu Rev Immunol (1994) 12:991–1045. doi: 10.1146/annurev.iy.12.040194.005015 8011301

[21] Matzinger P. The danger model: a renewed sense of self. Science (2002) 296:301–5. doi: 10.1126/science.1071059 11951032

[22] Bianchi ME. DAMPs, PAMPs and alarmins: all we need to know about danger. J Leukoc Biol (2007) 81:1–5. doi: 10.1189/jlb.0306164 17032697

[23] Amarante-Mendes GP Adjemian S Branco LM Zanetti LC Weinlich R Bortoluci KR. Pattern recognition receptors and the host cell death molecular machinery. Front Immunol (2018) 9:2379. doi: 10.3389/fimmu.2018.02379 30459758 PMC6232773

[24] Vénéreau E Ceriotti C Bianchi ME. DAMPs from cell death to new life. Front Immunol (2015) 6:422. doi: 10.3389/fimmu.2015.00422 26347745 PMC4539554

[25] Chen GY Nuñez G. Sterile inflammation: sensing and reacting to damage. Nat Rev Immunol (2010) 10:826–37. doi: 10.1038/nri2873 PMC3114424 21088683

[26] Yatim N Cullen S Albert ML. Dying cells actively regulate adaptive immune responses. Nat Rev Immunol (2017) 17:262–75. doi: 10.1038/nri.2017.9 28287107

[27] Muñoz LE Lauber K Schiller M Manfredi AA Herrmann M. The role of defective clearance of apoptotic cells in systemic autoimmunity. Nat Rev Rheumatol (2010) 6:280–9. doi: 10.1038/nrrheum.2010.46 20431553

[28] Galluzzi L Kepp O Chan FK-M Kroemer G. Necroptosis: Mechanisms and relevance to disease. Annu Rev Pathol Mech Dis (2017) 12:103–30. doi: 10.1146/annurev-pathol-052016-100247 PMC5786374 27959630

[29] Shaw PJ McDermott MF Kanneganti T-D. Inflammasomes and autoimmunity. Trends Mol Med (2011) 17:57–64. doi: 10.1016/j.molmed.2010.11.001 21163704 PMC3057120

[30] Schreiber A Rousselle A Becker JU von Mässenhausen A Linkermann A Kettritz R. Necroptosis controls NET generation and mediates complement activation, endothelial damage, and autoimmune vasculitis. Proc Natl Acad Sci (2017) 114:E9618–25. doi: 10.1073/pnas.1708247114 PMC5692554 29078325

[31] Chan J Roth J. Alarmins: awaiting a clinical response. J Clin Invest (2012) 122:2711–9. doi: 10.1172/JCI62423.The PMC3408740 22850880

[32] Jagiello P Gencik M Arning L Wieczorek S Kunstmann E Csernok E. New genomic region for wegener’s granulomatosis as revealed by an extended association screen with 202 apoptosis-related genes. Hum Genet (2004) 114:468–77. doi: 10.1007/s00439-004-1092-z 14968360

[33] Harper L Radford D Plant T Drayson M Adu D Savage COS. IgG from myeloperoxidase-antineutrophil cytoplasmic antibody-positive patients stimulates greater activation of primed neutrophils than IgG from proteinase 3-antineutrophil cytoplasmic antibody-positive patients. Arthritis Rheum (2001) 44:921–30. doi: 10.1002/1529-0131(200104)44:4<921::AID-ANR149>3.0.CO;2-4 11315931

[34] Abdgawad M Pettersson Å Gunnarsson L Bengtsson AA Geborek P Nilsson L. Decreased neutrophil apoptosis in quiescent ANCA-associated systemic vasculitis. PloS One (2012) 7:e32439. doi: 10.1371/journal.pone.0032439 22403660 PMC3293802

[35] Gabillet J Millet A Pederzoli-Ribeil M Tacnet-Delorme P Guillevin L Mouthon L. Proteinase 3, the autoantigen in granulomatosis with polyangiitis, associates with calreticulin on apoptotic neutrophils, impairs macrophage phagocytosis, and promotes inflammation. J Immunol (2012) 189:2574–83. doi: 10.4049/jimmunol.1200600 22844112

[36] Millet A Martin KR Bonnefoy F Saas P Mocek J Alkan M. Proteinase 3 on apoptotic cells disrupts immune silencing in autoimmune vasculitis. J Clin Invest (2015) 125:4107–21. doi: 10.1172/JCI78182 PMC4639994 26436651

[37] Kessenbrock K Krumbholz M Schönermarck U Back W Gross WL Werb Z. Netting neutrophils in autoimmune small-vessel vasculitis. Nat Med (2009) 15:623–5. doi: 10.1038/nm.1959 PMC2760083 19448636

[38] Xiao H Schreiber A Heeringa P Falk RJ Jennette JC. Alternative complement pathway in the pathogenesis of disease mediated by anti-neutrophil cytoplasmic autoantibodies. Am J Pathol (2007) 170:52–64. doi: 10.2353/ajpath.2007.060573 17200182 PMC1762697

[39] Wang H Wang C Zhao MH Chen M. Neutrophil extracellular traps can activate alternative complement pathways. Clin Exp Immunol (2015) 181:518–27. doi: 10.1111/cei.12654 PMC4557387 25963026

[40] Schreiber A Xiao H Jennette JC Schneider W Luft FC Kettritz R. C5a receptor mediates neutrophil activation and ANCA-induced glomerulonephritis. J Am Soc Nephrol (2009) 20:289–98. doi: 10.1681/ASN.2008050497 PMC2637051 19073822

[41] Söderberg D Segelmark M. Neutrophil extracellular traps in ANCA-associated vasculitis. Front Immunol (2016) 7:256. doi: 10.3389/fimmu.2016.00256 27446086 PMC4928371

[42] Martin KR Witko-Sarsat V. Proteinase 3: the odd one out that became an autoantigen. J Leukoc Biol (2017) 102:689–98. doi: 10.1189/jlb.3MR0217-069R 28546501

[43] Noronha IL Krüger C Andrassy K Ritz E Waldherr R. *In situ* production of TNF-alpha, IL-1 beta and IL-2R in ANCA-positive glomerulonephritis. Kidney Int (1993) 43:682–92. doi: 10.1038/ki.1993.98 8455368

[44] Irmscher S Brix SR Zipfel SLH Halder LD Mutlutürk S Wulf S. Serum FHR1 binding to necrotic-type cells activates monocytic inflammasome and marks necrotic sites in vasculopathies. Nat Commun (2019) 10:1–14. doi: 10.1038/s41467-019-10766-0 31273197 PMC6609651

[45] Schreiber A Pham CTN Hu Y Schneider W Luft FC Kettritz R. Neutrophil serine proteases promote IL-1β generation and injury in necrotizing crescentic glomerulonephritis. J Am Soc Nephrol (2012) 23:470–82. doi: 10.1681/ASN.2010080892 PMC3294298 22241891

[46] Tourneur L Witko-Sarsat V. Inflammasome activation: Neutrophils go their own way. J Leukoc Biol (2019) 105:433–6. doi: 10.1002/JLB.3CE1118-433R 30720889

[47] Kremserova S Nauseef WM. Frontline science: Staphylococcus aureus promotes receptor-interacting protein kinase 3- and protease-dependent production of IL-1β in human neutrophils. J Leukoc Biol (2019) 105:437–47. doi: 10.1002/JLB.4HI0918-346R PMC6927048 30548986

[48] Kerstein A Schüler S Cabral-Marques O Fazio J Häsler R Müller A. Environmental factor and inflammation-driven alteration of the total peripheral T-cell compartment in granulomatosis with polyangiitis. J Autoimmun (2017) 78:79–91. doi: 10.1016/j.jaut.2016.12.004 28040323

[49] Stegeman CA Tervaert JW Sluiter WJ Manson WL de Jong PE Kallenberg CG. Association of chronic nasal carriage of staphylococcus aureus and higher relapse rates in wegener granulomatosis. Ann Intern Med (1994) 120:12–7. doi: 10.7326/0003-4819-120-1-199401010-00003 8250451

[50] Laudien M Gadola SD Podschun R Hedderich J Paulsen J Reinhold-Keller E. Nasal carriage of staphylococcus aureus and endonasal activity in wegener s granulomatosis as compared to rheumatoid arthritis and chronic rhinosinusitis with nasal polyps. Clin Exp Rheumatol (2010) 28:51–5. 20412703

[51] Kitur K Parker D Nieto P Ahn DS Cohen TS Chung S. Toxin-induced necroptosis is a major mechanism of staphylococcus aureus lung damage. PloS Pathog (2015) 11:1–20. doi: 10.1371/journal.ppat.1004820 PMC4399879 25880560

[52] Greenlee-Wacker MC Rigby KM Kobayashi SD Porter AR DeLeo FR Nauseef WM. Phagocytosis of staphylococcus aureus by human neutrophils prevents macrophage efferocytosis and induces programmed necrosis. J Immunol (2014) 192:4709–17. doi: 10.4049/jimmunol.1302692 PMC4011196 24729616

[53] Churchill MEA Klass J Zoetewey DL. Structural analysis of HMGD-DNA complexes reveal influence of intercalation on sequence selectivity and DNA bending. J Mol Biol (2011) 403:88–102. doi: 10.1016/j.jmb.2010.08.031.Structural PMC2962916 20800069

[54] Rovere-Querini P Capobianco A Scaffidi P Valentinis B Catalanotti F Giazzon M. HMGB1 is an endogenous immune adjuvant released by necrotic cells. EMBO Rep (2004) 5:825–30. doi: 10.1038/sj.embor.7400205 PMC1299116 15272298

[55] Harris HE Andersson U Pisetsky DS. HMGB1: a multifunctional alarmin driving autoimmune and inflammatory disease. Nat Rev Rheumatol (2012) 8:195–202. doi: 10.1038/nrrheum.2011.222 22293756

[56] Henes FO Chen Y Bley TA Fabel M Both M Herrmann K. Correlation of serum level of high mobility group box 1 with the burden of granulomatous inflammation in granulomatosis with polyangiitis (Wegener’s). Ann Rheum Dis (2011) 70:1926–9. doi: 10.1136/ard.2010.146456 21765168

[57] Wibisono D Csernok E Lamprecht P Holle JU Gross WL Moosig F. Serum HMGB1 levels are increased in active wegener’s granulomatosis and differentiate between active forms of ANCA-associated vasculitis. Ann Rheum Dis (2010) 69:1888–9. doi: 10.1136/ard.2009.119172 20542962

[58] Bruchfeld A Wendt M Bratt J Qureshi AR Chavan S Tracey KJ. High-mobility group box-1 protein (HMGB1) is increased in antineutrophilic cytoplasmatic antibody (ANCA)-associated vasculitis with renal manifestations. Mol Med (2011) 17:29–35. doi: 10.2119/molmed.2010.00132 20844833 PMC3022976

[59] Manojlovic M Juto A Jonasdottir A Colic J Vojinovic J Nordin A. Microparticles expressing myeloperoxidase as potential biomarkers in anti-neutrophil cytoplasmic antibody (ANCA)-associated vasculitides (AAV). J Mol Med (2020) 98:1279–86. doi: 10.1007/s00109-020-01955-2 PMC7447662 32734361

[60] Wang C Wang H Chang D-Y Hao J Zhao M-H Chen M. High mobility group box 1 contributes to anti-neutrophil cytoplasmic antibody-induced neutrophils activation through receptor for advanced glycation end products (RAGE) and toll-like receptor 4. Arthritis Res Ther (2015) 17:1–13. doi: 10.1186/s13075-015-0587-4 25889374 PMC4382936

[61] Wang C Wang H Hao J Chang DY Zhao MH Chen M. Involvement of high mobility group box 1 in the activation of C5a-primed neutrophils induced by ANCA. Clin Immunol (2015) 159:47–57. doi: 10.1016/j.clim.2015.04.008 25934387

[62] Wang C Deng H Gong Y You R Chen M Zhao MH. Effect of high mobility group box 1 on toll-like receptor 9 in b cells in myeloperoxidase-ANCA-associated vasculitis. Autoimmunity (2020) 53:28–34. doi: 10.1080/08916934.2019.1696777 31790283

[63] Wang C Chang D-Y Chen M Zhao M-H. HMGB1 contributes to glomerular endothelial cell injury in ANCA-associated vasculitis through enhancing endothelium-neutrophil interactions. J Cell Mol Med (2017) 21:1351–60. doi: 10.1111/jcmm.13065 PMC5487910 28181422

[64] Page TH Chiappo D Brunini F Garnica J Blackburn J Dudhiya F. Danger-associated molecular pattern molecules and the receptor for advanced glycation end products enhance ANCA-induced responses. Rheumatol (United Kingdom) (2022) 61:834–45. doi: 10.1093/rheumatology/keab413 PMC8824420 33974049

